# Incidental Small Cell Neuroendocrine Carcinoma and High-Grade Dysplasia in an Orthotopic Ileal Neobladder Three Decades Post-cystectomy: A Case Report

**DOI:** 10.7759/cureus.115171

**Published:** 2026-08-25

**Authors:** Gabriana M Farah, Gabrielle R Yankelevich, Becky Madory, Benjamin V Stone

**Affiliations:** 1 School of Medicine, Rush University, Chicago, USA; 2 Department of Urology, Medical University of South Carolina, Charleston, USA; 3 Department of Pathology, Ralph H. Johnson Veteran Affairs Medical Center, Charleston, USA

**Keywords:** high-grade dysplasia, orthotopic neobladder, prostatic adenocarcinoma, small cell neuroendocrine carcinoma, urinary diversion

## Abstract

The utilization of a bowel diversion is standard following radical cystectomy for muscle-invasive bladder cancer. Neoplastic transformations are a known long-term complication of intestinal urinary diversions, although uncommon. Most documented tumors are adenocarcinomas, with neuroendocrine tumors present in exceedingly rare instances. To the best of our knowledge, we present the second case of neuroendocrine tumor found in an orthotopic neobladder. We review the workup and treatment as well as our proposed treatment for low- versus high-risk lesions within a neobladder. This case stresses the importance of lifelong, close monitoring of patients with intestinal urinary diversions.

## Introduction

Bowel diversions are frequently used for reconstruction after cystectomy to allow a patient to maintain high functionality [1,2]. Orthotopic neobladders, or ileal neobladder, are commonly used as they allow for normal voiding habits, maintenance of continence, and preservation of the upper urinary tract anatomy [1]. However, a well-documented, though uncommon, long-term complication is the development of neoplasia within the substituted bowel segment [2,3].

While intestinal urinary diversions are associated with a relatively low overall risk of secondary malignancies when compared to other diversions, such as ureterosigmoidostomies, the range of tumors that can arise in isolated intestinal diversions is vast [4]. These can include urothelial carcinoma, squamous cell carcinoma, signet ring cell carcinoma, and adenocarcinoma [4,5]. Well-differentiated neuroendocrine tumors (NETs) have also been reported but are exceptionally rare [6]. To the best of our knowledge, we present the fourth case of NET after ileal diversion and the second case of NET after orthotopic ileal neobladder [6,7].

We present the unique case of an 81-year-old man who, over 30 years after his initial radical cystectomy, developed rapidly progressing high-grade dysplasia in his orthotopic ileal neobladder and whose final surgical pathology unexpectedly revealed a poorly differentiated small cell neuroendocrine carcinoma. Due to these secondary tumors being so rare, there are no standard protocols regarding their surveillance and management [6]. Therefore, we also propose a pathway for management of low-grade dysplasia, high-grade dysplasia, and aberrant pathology.

## Case presentation

The patient is an 81-year-old male who presented to the urology clinic for evaluation of gross hematuria and multidrug-resistant recurrent urinary tract infections while on daily prophylactic nitrofurantoin. His urologic history is significant for muscle-invasive bladder cancer, for which he underwent a radical cystectomy and orthotopic neobladder creation in the 1990s, where he was told that a small portion of his prostate was left in situ at the time of the surgery. He subsequently developed prostate cancer in the residual prostate tissue and underwent radiation therapy in March 2025. The patient’s other medical history includes hyperlipidemia, chronic kidney disease, hypertension, hypothyroidism, right bundle branch block, obesity (BMI 33.6), hypogonadism, chronic diarrhea, restless leg syndrome, anemia, heart failure with preserved ejection fraction, and post-traumatic stress disorder.

Physical examination upon initial presentation was unremarkable, with a soft, non-tender abdomen, intact surgical scars, and no palpable suprapubic masses or costovertebral angle tenderness. He underwent cystoscopy in clinic, which showed several polypoid lesions along the left posterior neobladder wall and small bladder neck contracture. Biopsies were obtained demonstrating small intestinal mucosa with low-grade adenomatous dysplasia. These lesions were fulgurated, and gastroenterology was consulted and recommended a three-month follow-up cystoscopy. Unfortunately, after the first biopsy, the patient reported new onset of severe stress urinary incontinence despite frequent catheterization.

At his three-month repeat cystoscopy, repeat biopsies again demonstrated small intestinal mucosa with low-grade adenomatous dysplasia found on the left posterior and lateral neobladder walls. These lesions were again fulgurated and in discussion with gastroenterology, he was planned for a six-month follow-up cystoscopy. Unfortunately, at his six-month surveillance cystoscopy, the lesions had grown to over 5 cm on the left lateral and posterior neobladder walls. The ileal chimney was noted to have moderate length (~10 cm estimated on flexible cystoscopy) and no involvement with tumors. The specimen was pathologically confirmed to be high-grade dysplasia. Due to the risk of neobladder perforation if the entire mass was to be endoscopically resected, a visually incomplete resection was performed. 

**Figure 1:**
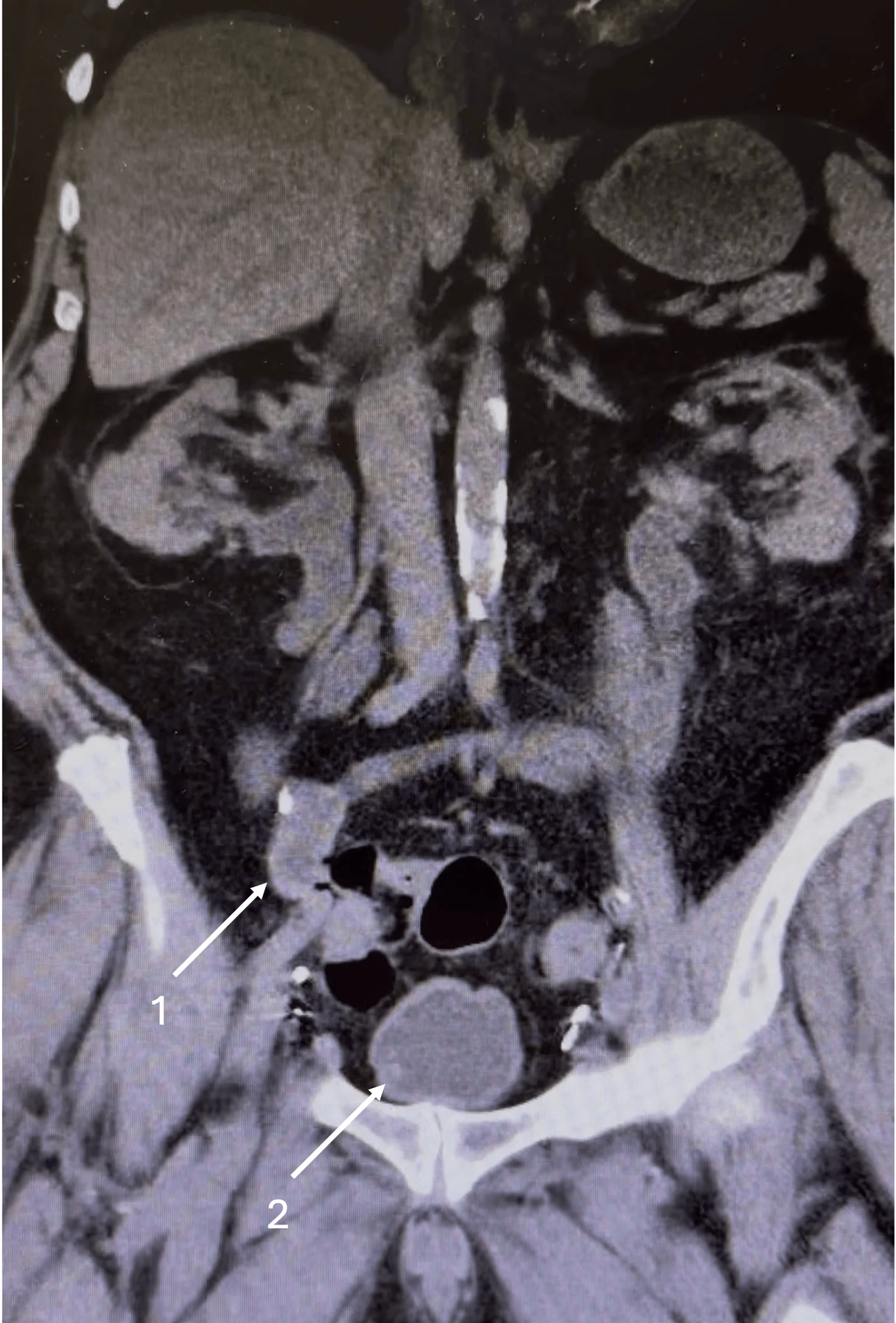
Coronal CT imaging showing ileal chimney (1) and neobladder (2).

**Figure 2:**
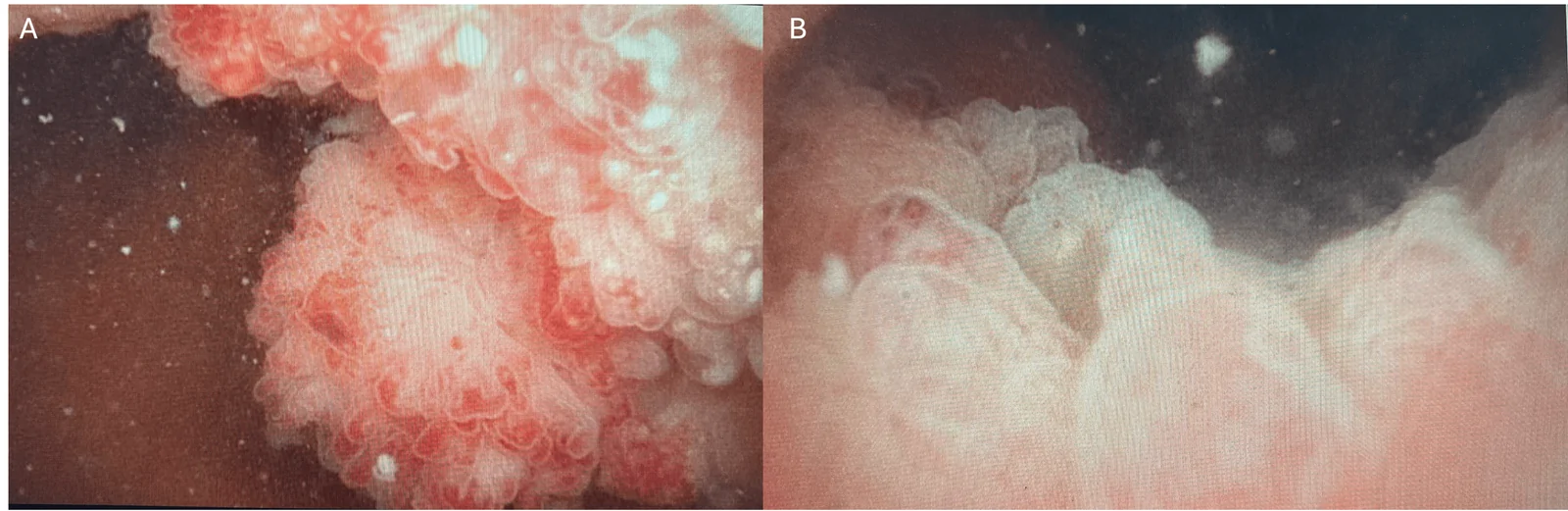
(A, B) Preoperative cystoscopic images in neobladder showing tumor burden.

The case was discussed in a multidisciplinary fashion with urology, gastroenterology, and colorectal surgery. Given the rapid progression from low-grade to high-grade dysplasia, as well as high-volume urinary incontinence since the first endoscopic procedure, he was planned for open neobladder removal and conversion to ileal conduit. He had preoperative imaging (Figure 1) showing a good length of the ileal chimney, as well as cystoscopy confirming ileal chimney length of about 7 cm. Figure 2 shows the preoperative cystoscopy with tumors present within the neobladder. 

In the operating room, a lower midline abdominal incision was made and access was gained to the extraperitoneal cavity where extensive lysis of adhesions was performed to identify the neobladder. Anterior dissection was performed under the pubic bone to the neobladder-urethral anastomosis and the neobladder was circumferentially dissected off the urethra, leaving the prostate in situ. The ileal chimney was measured intraoperatively with length ~10cm and was dissected off the neobladder to allow complete excision of the neobladder. Lastly, to reconstruct the urinary tract, the team utilized the existing ileal chimney and converted it into an ileal conduit.

**Figure 3:**
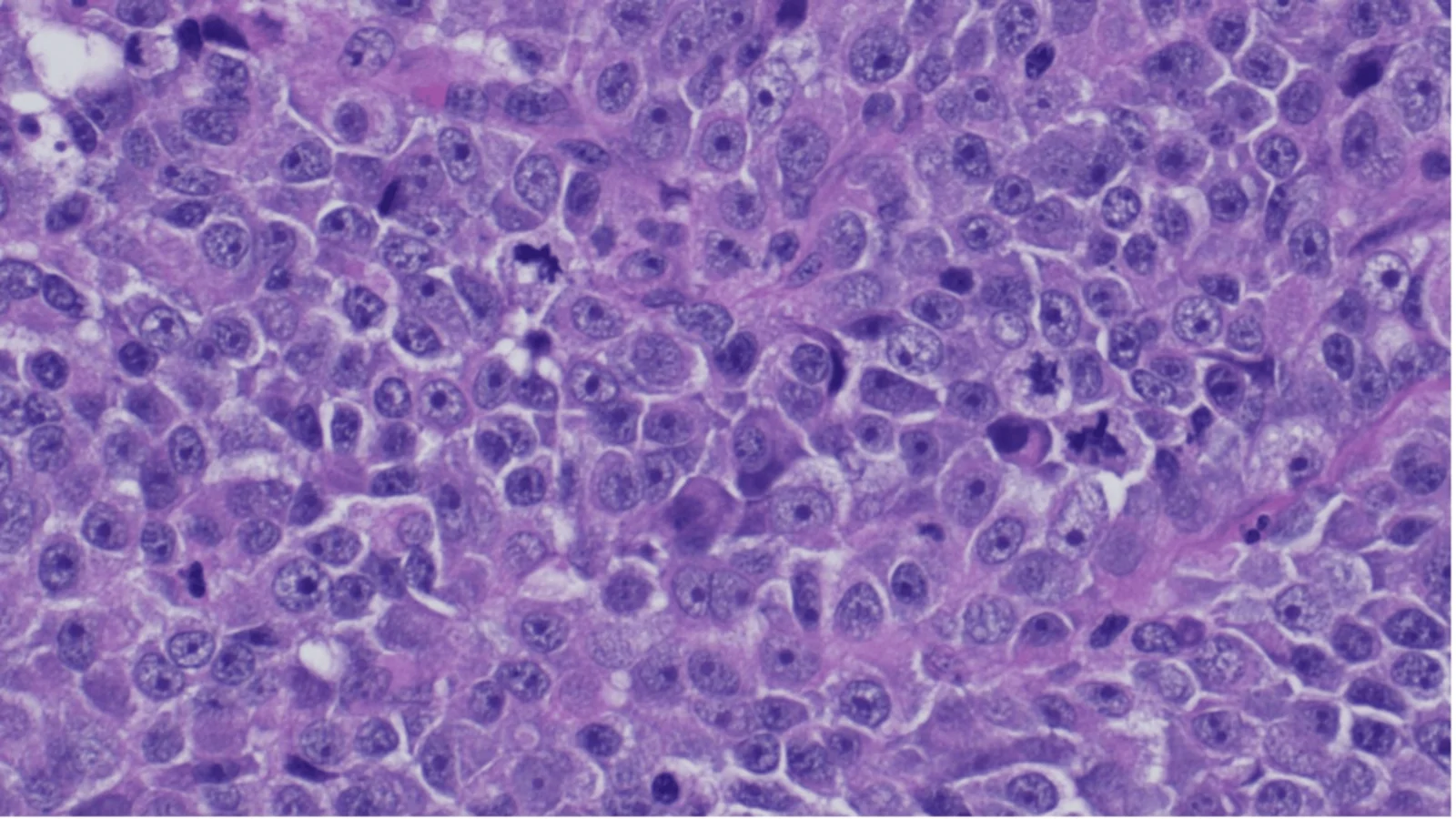
Poorly differentiated small cell neuroendocrine carcinoma. H&E stain at 40X magnification taken from a 0.4 cm (stage pT1a) polyp. The image reveals aggressive, grade 3 cytologic features with multiple mitotic figures, indicative of a highly proliferative malignancy (Ki-67 index > 80%).

**Figure 4:**
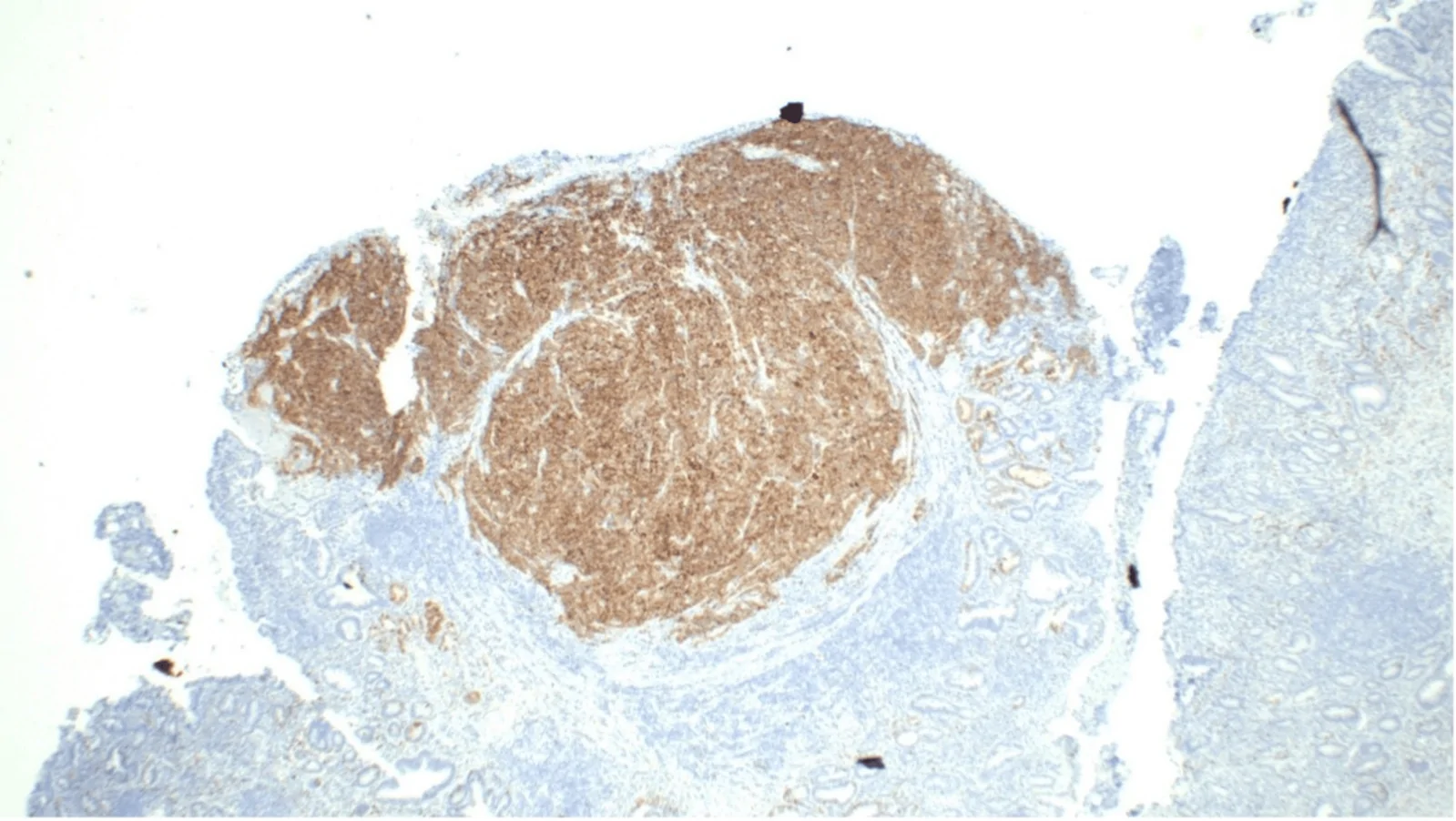
Immunohistochemical staining of the small cell neuroendocrine carcinoma. The tumor cells, arising from a 0.4 cm focus (stage pT1a), exhibit diffuse, strong positivity for synaptophysin at 2X magnification, confirming neuroendocrine differentiation.

**Figure 5:**
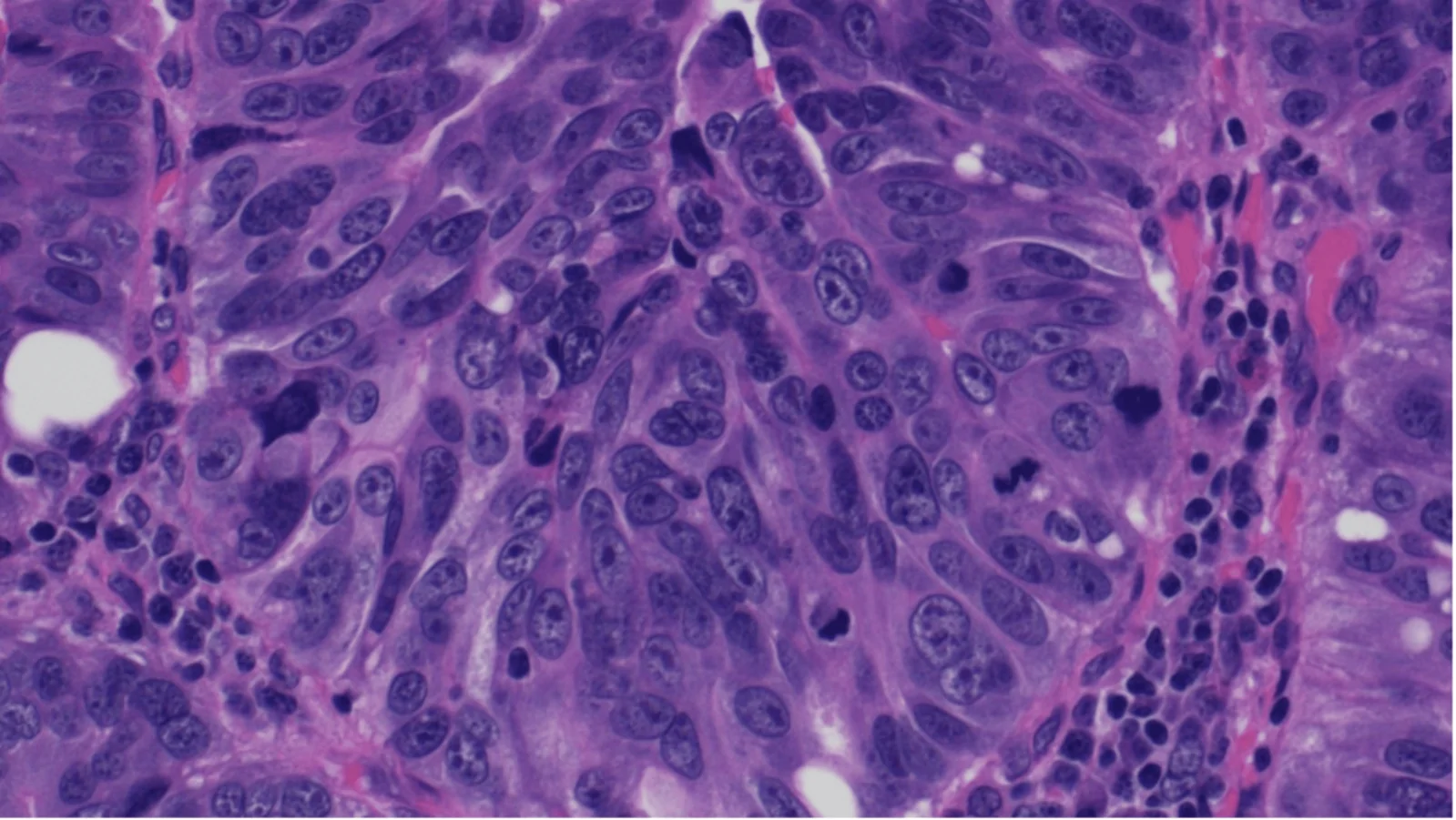
High-grade dysplasia of the glandular epithelium. H&E stain at 40X magnification taken from a 0.2 cm mucosal polyp within the neobladder. The image demonstrates marked cytologic atypia, nuclear enlargement, and architectural distortion without evidence of lamina propria invasion.

The final surgical pathology of the explanted neobladder revealed the presence of grade 3, poorly differentiated pT1a small cell neuroendocrine carcinoma, measuring 0.4 cm (Figure 3). Immunohistochemical staining of this first polyp was positive for AE1/AE3, chromogranin, synaptophysin (Figure 4), and INSM1, with a Ki-67 stain showing greater than 80% nuclear positivity. Pathology also confirmed the presence of high-grade dysplasia of the glandular epithelium without evidence of invasion (Figure 5), and lacking neuroendocrine differentiation, staining negative for INSM1, with a Ki-67 index of approximately 50%. Additional findings included surface disruptions of the neobladder which exposed small intestinal mucosa with underlying low-grade adenomatous dysplasia. All surgical margins were negative for invasive carcinoma.

Postoperatively, the patient’s diet was advanced to regular by postoperative day two after having a bowel movement. His creatinine had peaked postoperatively to 2.8 mg/dL, but improved with increased intravenous fluids and ultimately decreased to 2.09 mg/dL on postoperative day three (patient’s baseline ~2.2 mg/dL). He was discharged on postoperative day three. He was sent to oncology and, after a negative PET scan, he underwent four cycles of carboplatin/etoposide, with a plan for follow-up in three months. 

## Discussion

While the development of malignant or highly dysplastic lesions within urinary diversions utilizing bowel segments is uncommonly seen, it is a well-known long-term risk of the procedure [2]. Aside from recurrent urothelial carcinoma, the specific neoplastic growths that have been identified in the segments of bowel used for urinary diversions mainly include adenomatous lesions of tubular, tubulovillous, and villous histologies [8,9]. Despite these being benign growths, they do carry a risk of malignant transformation, particularly into enteric-type adenocarcinoma (EA) [3,5]. The development of an invasive lesion within a neobladder, whether a high-grade dysplastic adenoma or EA, occurs following a long latency period [5]. Retrospective data have shown that the latency period between the urinary diversion surgery and this development can range from four to 32 years [3,9].

The pathogenesis of carcinogenesis within these translocated intestinal segments is still unclear, however there is a multifactorial hypothesis. First, it substantiates that the continuous contact between urine and the urointestinal mucosa leads to chronic inflammation and mucosal atrophy [3,9]. In addition, the bacterial colonization within the intestinal diversion introduces hydrolytic enzymes which may activate specific carcinogens in the intestinal mucosa. Specifically, they have the capacity to convert nitrates in the urine into nitrosamines, a reaction which is facilitated by the distinct urinary pH inside the neobladder [4]. High levels of potentially carcinogenic nitrosamines have been found in the urine of patients following ileal cystoplasty, levels that have been found to be significantly higher than those found in normal controls [4,9]. These factors, when joined together, induce mucosal instability and promote oncogenesis [9].

Secondary tumors arising in isolated intestinal segments may have highly variable histologies, however most present as adenomatous lesions [5,9]. The literature points to adenocarcinoma arising from precursor lesions, mainly tubular or tubulovillous adenomas with high-grade dysplasia [5,8]. This case presents a uniquely rare deviation from this proposed sequence by depicting an incidental small cell neuroendocrine carcinoma. Small intestinal NETs arise from intraepithelial endocrine cells, and presenting within an ileal neobladder is exceptionally rare [6,7]. There have been previously reported cases describing well-differentiated NETs in neobladders; however, our patient exhibited a highly aggressive, poorly differentiated small cell carcinoma with a Ki-67 index exceeding 80% [6,7]. Small cell carcinoma is a rare variant of NET itself, which is characterized by aggressive progression, and reports of it being found in an orthotopic urinary diversion are exceedingly scarce [10]. Therefore, we add to this rarity by presenting, to the best of our knowledge, the fourth case of NET after ileal diversion and the second case of NET after orthotopic ileal neobladder [6,7].

The latency period involved in this case is quite consistent with the existing literature reporting secondary neoplasms in neobladders [3,5]. While recurrent urothelial carcinomas present within the first few years post-cystectomy, primary enteric malignancies and secondary tumors in neobladders have a latency period of several decades [5,9]. Due to primary neoplastic changes of the ileum being rare, documentation of late developments of bowel segment tumors in urinary diversions highlight the necessity for close, long-term follow-up [8]. It has been proposed that patients with translocated bowel in their urinary tracts undergo continuous endoscopic and radiologic surveillance beginning 10 years after their initial cystectomy [4,9].

This approach avoided the morbidity associated with creating a new bowel anastomosis or ureteral anastomoses. Conversion from orthotopic neobladder to ileal conduit has been reported for several prototype scenarios including oncologic failure, functional failure, and renal deterioration. Oncologic failure arises when a patient has a urethral recurrence or occurrence within the neobladder. Urethral recurrence was reported by Freeman et al. to be at 7.9% at five years [11]. Urethral recurrences can be treated with transurethral resection, urethrectomy with conversion of the neobladder to continent catheterizable diversion or conduit, or chemotherapy [12-14]. Moreover, Collins and colleagues looked at their hospital system for pathologic specimens between 2002 and 2022 to identify malignancy in ileal conduits or orthotopic neobladders [15]. They found a total of 13 patients who had malignancy with 10 having recurrent urothelial carcinoma and three having a new primary adenocarcinoma [15]. Next, conversion to an ileal conduit can be performed for functional failure including retention or inability to catheterize [16]. Furthermore, progressive renal impairment is an indication for conversion to an ileal conduit, with Zahran et al. reporting 41% of patients in their female cohort developed chronic kidney disease stage III to IV [16,17].

The initial challenge in this case was in managing the local progression of the disease. Given there are no specific guidelines for this scenario, our gastroenterology colleagues used the National Comprehensive Cancer Network (NCCN) guidelines for small and large bowel. NCCN guidelines for surveillance recommend a similar approach to colorectal surveillance due to a lack of small bowel adenocarcinoma-specific data [18]. The American Gastroenterological Association recommends surveillance for small adenomas < 10mm with low-grade dysplasia with a repeat colonoscopy within seven to 10 years given low risk for advanced neoplasia [19]. However, patients with five to 10 tubular adenomas < 10 mm or one or more adenomas > 10 mm should undergo repeat colonoscopy in three years as the risk for advanced neoplasia increases [19]. However, NCCN guidelines for colon cancer recommend colectomy for unfavorable pathology which includes high-grade histology, depth of submucosal invasion > 1 mm, high tumor budding, or positive margins [20]. Given these guidelines, in conjunction with bladder cancer surveillance guidelines, for low-grade dysplasia we propose surveillance cystoscopy every three months for the first year, every six months for year two, and then annually thereafter. For high-grade dysplasia, we recommend removal of the ileal component with the dysplasia. If the ileal chimney is not involved, such as in our patient, then we can reasonably convert to an ileal conduit.

## Conclusions

The utilization of the small intestine for urinary tract diversion carries a low but clear risk for the future development of secondary neoplasms. With a decades-long latency period, patients with these intestinal urinary diversions require lifelong surveillance. As this report demonstrates, endoscopic biopsies may demonstrate low-grade and high-grade dysplasia, but these could be masking co-existing, highly aggressive malignancies including small cell neuroendocrine carcinoma. The unpredictable and aggressive nature of neoplastic changes in a neobladder emphasizes the necessity for prompt clinical recognition and definitive surgical excision when endoscopic management fails to halt morphological progression.
